# Multi-omic analysis unveils biological pathways in peripheral immune system associated to minimal hepatic encephalopathy appearance in cirrhotic patients

**DOI:** 10.1038/s41598-020-80941-7

**Published:** 2021-01-21

**Authors:** Teresa Rubio, Vicente Felipo, Sonia Tarazona, Roberta Pastorelli, Desamparados Escudero-García, Joan Tosca, Amparo Urios, Ana Conesa, Carmina Montoliu

**Affiliations:** 1 Laboratory of Neurobiology, Centro Investigación Príncipe Felipe, Valencia, Spain; 2 Departamento de Estadística e Investigación Operativa Aplicadas y Calidad, Universitat Politècnica de València, Valencia, Spain; 3 Protein and Metabolite Biomarkers Unit, Laboratory of Mass Spectrometry, Istituto di Ricerche Farmacologiche Mario Negri IRCCS, Milan, Italy; 4 Unidad de Digestivo, Hospital Clínico de Valencia, Departamento Medicina, Universidad de Valencia, Valencia, Spain; 5 Unidad de Digestivo, Hospital Clínico de Valencia, Valencia, Spain; 6 Neurological Impairment Laboratory, Fundación Investigación Hospital Clínico Universitario de Valencia, Instituto de Investigación Sanitaria-INCLIVA, Valencia, Spain; 7 Microbiology and Cell Science Department, Institute for Food and Agricultural Sciences, Genetics Institute, University of Florida, Gainesville, USA; 8 Departamento de Patología, Facultad de Medicina, Universidad de Valencia, Valencia, Spain

**Keywords:** Neuroimmunology, Data integration

## Abstract

Patients with liver cirrhosis may develop minimal hepatic encephalopathy (MHE) which affects their quality of life and life span. It has been proposed that a shift in peripheral inflammation triggers the appearance of MHE. However, the mechanisms involved in this immune system shift remain unknown. In this work we studied the broad molecular changes involved in the induction of MHE with the goal of identifying (1) altered genes and pathways in peripheral blood cells associated to the appearance of MHE, (2) serum metabolites and cytokines with modified levels in MHE patients and (3) MHE-regulated immune response processes related to changes in specific serum molecules. We adopted a multi-omic approach to profile the transcriptome, metabolome and a panel of cytokines of blood samples taken from cirrhotic patients with or without MHE. Transcriptomic analysis supports the hypothesis of alternations in the Th1/Th2 and Th17 lymphocytes cell populations as major drivers of MHE. Cluster analysis of serum molecules resulted in six groups of chemically similar compounds, suggesting that functional modules operate during the induction of MHE. Finally, the multi-omic integrative analysis suggested a relationship between cytokines CCL20, CX3CL1, CXCL13, IL-15, IL-22 and IL-6 with alteration in chemotaxis, as well as a link between long-chain unsaturated phospholipids and the increased fatty acid transport and prostaglandin production. We found altered immune pathways that may collectively contribute to the mild cognitive impairment phenotype in MHE. Our approach is able to combine extracellular and intracellular information, opening new insights to the understanding of the disease.

## Introduction

Minimal hepatic encephalopathy (MHE) is a neuropsychiatric syndrome that produces mild cognitive impairment, attention deficits, psychomotor slowing and impaired coordination in cirrhotic patients^1^. The combination of hyperammonemia and peripheral inflammation, even before progression to cirrhosis, is enough to induce cognitive and motor disturbances^2^. Specific alterations of the immunophenotype have been already described as triggers of MHE in cirrhotic patients^3^. Furthermore, infiltration of peripheral lymphocytes in cerebellum has been observed at early stages of liver disease^4^. This suggests that changes in peripheral inflammation cause infiltration of lymphocytes in the brain, leading to neuroinflammation which alters neurotransmission and ultimately leads to cognitive impairment^5^. The neuro-immune axis has been found to be relevant in many other neurological human diseases such as multiple sclerosis^6^, hepatocellular carcinoma^7^, Alzheimer’s disease and other neurological disorders^8^. Potential pro-inflammatory biomarkers have been suggested, including chemokines, which mediate acute inflammation by driving leukocyte migration to damaged or infected tissues^6^. Beyond their classical chemotactic functions, these immune proteins have been implicated in different neural processes such as neuromodulation, neurotransmission and regulation of neurogenesis^8^.

The precise pathways that trigger a systemic immune response leading to a neurological disorder are still poorly understood. Next to the role of pro-inflammatory chemokines, other signaling, metabolic and regulatory components are likely to contribute. Here, we have adopted a multi-omic approach to investigate the set of molecular and cellular events that are associated with MHE. Multi-omic technologies such as genomics, epigenomics, transcriptomics, proteomics and metabolomics are increasingly being used to profile the multi-layered component of living cells and pathological processes^9–12^. The rationale behind this strategy is that disease usually impacts different types of biomolecules and by expanding the molecular space under study, the likelihood of identifying relevant biomarkers will increase. In the context of neurological pathologies, multi-omics has allowed the modeling of the highly sophisticated brain metabolic networks and their contribution to human health^13,14^, as well as the identification of specific and common features for this type of diseases^15^.

In this study we combine blood transcriptomics, serum metabolomics and cytokines to identify pathways and biomarkers associated with MHE in cirrhotic patients. We deploy a bioinformatics analysis pipeline that combines univariate, multivariate and enrichment methods to find genes and extracellular compounds that share variation patterns linked to the MHE phenotype. Using this approach, we discovered a relationship between extracellular CCL20, CX3CL1, CXCL13, IL-15, IL-22 and IL-6 with the alteration of chemotactic receptors and ligands in MHE patients. In addition, this methodology suggested a link between long-chain unsaturated phospholipids and the increment of fatty acid transport and prostaglandin production, which jointly may contribute to the onset of mild cognitive impairment.

## Methods

### Overview of the analysis strategy

**Figure 1:**
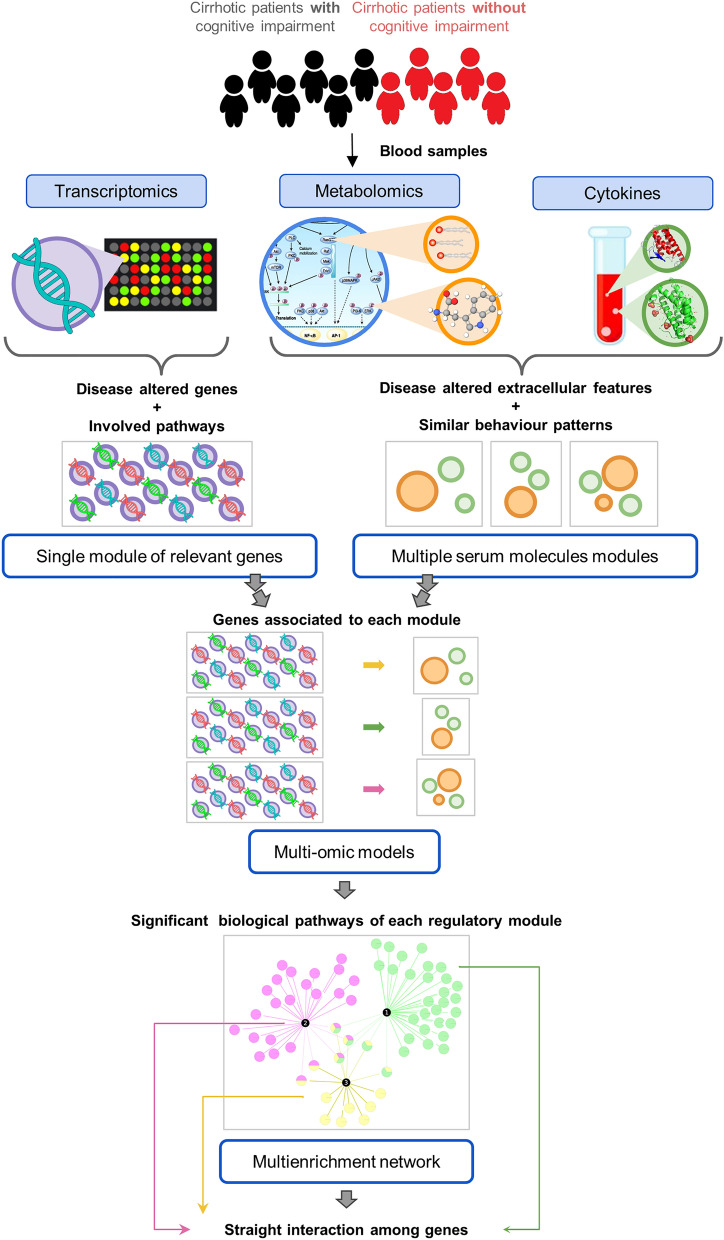
Summary of the analysis pipeline. Transcriptomics, metabolomics and cytokines were measured in blood samples of 11 cirrhotic patients (6 with and 5 without MHE). Step 1: genes and pathways altered in MHE. Step 2: altered metabolites and cytokines grouped by correlation. Step 3: integration of extracellular and intracellular datasets. Step 4: enrichment and network analysis of iterative models.

In order to understand the joint contribution of gene expression and extracellular metabolite changes to MHE, we developed an extensive pipeline that combines univariate and multivariate regression for feature selection while taking into account the biology of the system (Fig. 1). Basically, we first identified genes and associated pathways that significantly changed between cirrhotic individuals with and without MHE. In parallel, metabolites and cytokines with significant serum level changes were identified and grouped by their variation pattern across individuals. These groups represent metabolic modules that change in a coordinated fashion. Next, we asked if there exists a gene expression signature that associates to each of these modules. We applied Partial Least Square (PLS) to the multi-omic data taking the selected genes as explanatory variables and each module of extracellular compounds as a shared response. This analysis selected the genes with the strongest associations to each metabolic group, which are then further investigated by pathway enrichment and biological interaction modeling (Fig. 1).

### Patients and sample collection

11 patients with liver cirrhosis were recruited from the outpatient clinics in the Hospital Clínico of Valencia, Spain. The diagnosis of cirrhosis was based on clinical, biochemical and ultrasonographic data. Exclusion criteria were: overt hepatic encephalopathy, recent (< 6 months) alcohol intake, infection, recent (< 6 weeks) antibiotic use or gastrointestinal bleeding, recent (< 6 weeks) use of drugs affecting cognitive function, presence of hepatocellular carcinoma, or neurological or psychiatric disorder. Patients included in the study did not show fever or any clinical or biological sign of recent infection. None of the patients included in the study had hypothyroidism or altered TSH (Thyroid-Stimulating Hormone). All participants were included in the study after signing a written informed consent. Study protocols were approved by the Scientific and Ethical Committees of the Hospital Clínico of Valencia. The procedures followed were in accordance with the ethical guidelines of the Declaration of Helsinki^3^.

#### Diagnosis of MHE

MHE was diagnosed using the Psychometric Hepatic Encephalopathy Score (PHES) which comprises 5 psychometric tests^16,17^. The scores were adjusted for age and education level using Spanish normality tables (www.redeh.org). Patients were classified as MHE when the score was ≤ − 4 points. From the 11 recruited cirrhotic patients, five were diagnosed without MHE and six with MHE. All patients were males and the mean age in group with MHE (70.7 ± 4.0) and in group without MHE (63.8 ± 1.2) did not show significant differences (p = 0.157).

#### Sample collection

Transcriptomic dataset was measured in blood samples collected in PAXgene^®^ blood RNA tubes (BD Biosciences) that were frozen at − 20 °C during 24 h and kept at − 80 °C for subsequent analysis. For metabolomic measurements, plasma samples were obtained from blood collected in BD P100 tubes (BD Biosciences) containing EDTA and protein stabilizers. Plasma samples were obtained after two serial centrifugations, first at 5 °C, 2500*g*, for 20 min, and after transferring plasma to new tubes, a second centrifugation at 10 °C, 2500*g*, for 10 min, were performed. Plasma samples were distributed in several aliquots and stored at − 80 °C. Cytokines were measured in serum fraction after blood sample collection in tubes without EDTA.

### Analysis of cytokines in serum samples

Concentration of IL-4, IL-6, IL-13, IL-17, IL-18, IL-22, TGFβ (Affymetrix eBioscience, Vienna, Austria), IL-10, IL-12, IL-15, CXCL13, CCL20, CX3CL1 and TNFα (R&D Systems, Minneapolis, MN, USA) were measured by ELISA according to the manufacturer’s instructions. High sensitivity kits were necessary for IL-17, IL-4 and TNFα assessment.

#### Cytokines data analysis

Significant differences in cytokines between patients with and without MHE were tested using the non-parametric Wilcoxon test after homoscedasticity were checked. Multiple testing correction was applied by FDR.

### Transcriptomic profiling of plasma samples

RNA extracted from peripheral blood cells was quantified with NanoDrop ND1000 (NanoDrop Technologies, Wilminton, Delaware USA) and RNA quality was confirmed with RNA 6000 Nano Bioanalyzer assay (Agilent Technologies, Palo Alto, California USA). Using the One-Color Low Input Quick Amp Labelling Kit (Agilent p/n 5190-2305) according to the manufacturer’s instructions, 200 ng of total RNA were used to produce Cyanine 3-CTP-labeled cRNA. Following ‘One-Color Microarray-Based Gene Expression Analysis’ protocol Version 6.7 (Agilent p/n G4140-90040), 600 ng of labeled cRNA was hybridized with the SurePrint G3 Human Gene Expression Microarrays v3 8X60K (Agilent p/n G4858A-072363). Microarrays were scanned in an Agilent Microarray Scanner (Agilent G2565C) as reported in the guidelines from the manufacturer and the image analysis was made with Agilent Feature Extraction Software 11.5.1.1 using default parameters (protocol GE1_1105_Oct12, grid template 072363_D_F_20150612 and QC Metric Set GE1_QCMT_Oct12).

#### Transcriptomic data analysis

The normalization method VSN, implemented in the **vsn** R/Bioconductor package^18^, was applied to stabilize the variance of microarray intensity data. Weighted linear modeling from **limma** R/Bioconductor package^19^ was performed to find differentially expressed genes between two groups: cirrhotic patients with and without cognitive impairment. Enrichment analysis was calculated using Fisher’s Exact Test implemented in PaintOmics^20^, which identifies those gene sets that share an unusually large number of genes within the list resulting from the differential expression test. PaintOmics includes KEGG database^21^ as annotation for enrichment analyses and subsequent gene expression representation.

### Metabolomic profiling of serum samples

Targeted metabolomic analysis was performed using the Absolute-IDQ™ P180 kit (BIOCRATES Life Sciences AG, Innsbruck, Austria). The metabolite extracts were processed following the instructions by the manufacturer and analyzed on a triple-quadropole mass spectrometer (AB SCIEX triple-quad 5500) operating in the multiple reaction monitoring (MRM-MS) mode. The assay is based on PITC (phenylisothiocyanate)-derivatization in the presence of internal standards for the analysis of aminoacids and biogenic amines resolved and analyzed by liquid chromatography (LC) tandem mass spectrometry (MS/MS) using scheduled MRMs. Separation was achieved on a Agilent Zorbax Eclipse XDB C18 column (3 × 100 mm, 3.5 μm) with a mobile phases of 0.2% formic acid in water (A) and 0.2% formic acid in acetonitrile (B). The gradient program was as follows: 0 min, 100% A; 0.5 min, 100% A; 5.5 min, 5% A; 6.5 min, 5% A; 7.0 min, 100% A; 9.5 min 100% A. The column was maintained at 50 °C, with 0.5 ml/min flow rate and 10 μl volume injection. In the mass spectrometer, sample analysis was performed in positive electrospray ionization (ESI) mode. Declustering potential (DP) and collision energy (CE) were provided by the kit. The MRM MS/MS detector conditions were set as follows: temperature 500 °C; ion spray voltage 5500 V; curtain gas 20 psi; collision gas (CAD) medium; entrance potential (EP) 10 V; collision cell exit potential (CXP) 15 V; ion source gas 1 (GS1) 40 psi; ion source gas 2 (GS2) 50 psi. Retention times for every metabolite were adjusted previously to the experiment^22^.

To analyze acylcarnitines, glycerophospholipids, hexose, subsequent flow injection analysis tandem mass spectrometry (FIA-MS/MS) was performed. PEEK Tubing Red (1/16 × 0.005) was used for separation with Biocrates solvent I diluted in 290 ml methanol as mobile phase ("isocratic elution mode"). The gradient program: 0 min, 0.03 ml/min; 1.6 min, 0.03 ml/min; 2.4 min, 0.20 ml/min; 2.8 min, 0.20 ml/min; 3.0 min, 0.03 ml/min. Positive electrospray ionization (ESI) mode was used in spectrometry although some parameters were different for lipids and sugars. For lipids, exactly the same parameters used in LC–MS/MS except the temperature (200 °C). For sugars: ion spray voltage − 4500 V (negative); curtain gas 20 psi; CAD medium; EP − 10 V; CXP − 15 V; GS1 40 psi; GS2 50 psi; DP − 55 V; CE − 12 V^22^.

Lipid concentrations were automatically calculated in μM using the MetIDQTM software (Biocrates Life Science AG, Innskruck, Austria) and the rest of metabolites were quantified from standard curves with the software Analyst from SCIEX. Isotope-labeled internal standards are integrated into the platform for metabolite absolute quantification. The measurements are made in a 96-well format. Metabolite limit of detection was set three times the value of the “zero samples” and the average coefficient of variation of the metabolites among the biological replicates was 30%. Based on the five internal quality controls, technical variation was below 15%. A metabolite was excluded from further analyses if its concentration measurement data did not meet all of the following criteria: (1) minor of 20% of missing values (non-detectable peak) for each quantified metabolite in each experimental group (2) 50% of all measured sample concentrations for the metabolite had to be above the limit of detection^22^. The provided metabolomic data matrix included 143 metabolites without missing values: 8 acylcarnitines, 21 amino acids, 17 biogenic amines, the sum of hexoses, 15 sphingomyelins (SM) and 81 glycerophospholipids, including lysophosphatidylcholines (lysoPC) and phosphatidylcholines (PC). According platform annotation, lipid side-chain composition is abbreviated as “C x:y”, where “x” denotes the number of carbon atoms and “y” the number of double bonds present in the fatty acid residues. The presence of a hydroxyl group in SMs is indicated with OH. Glycerophospholipids are differentiated according to the presence of ester and ether bonds in the glycerol moiety. Double letters (aa = diacyl, ae = acyl-alkyl) indicate that two glycerol positions are bound to a fatty acid residue, while a single letter (a = acyl or e = alkyl) indicates a bond with only one fatty acid residue^23^.

#### Metabolomic data pre-processing and analysis

As in transcriptomics, **vsn** normalization^18^ and **limma**^19^ procedures were applied to find the altered metabolites between groups of patients. Significantly altered metabolites in MHE were considered when FDR < 0.05. Although **vsn** and **limma** were primarily developed for microarray data, they can be applied to mass spectrometry-based data since normality assumption holds. The effectiveness for using these methods in metabolomic datasets has been already validated in different studies^24–28^.

### Omic power analysis

The statistical power of each omic data type was evaluated with the MultiPower tool^29^ as a quality control of differential expression results. For gene expression and metabolomic data, we found values of power above 0.75 when aiming to detect features with relatively large changes and low variability (Cohen’s d of at least 1.5). For cytokines, a power of at least 0.75 was obtained for a higher proportion of features since they present bigger effect sizes compared to the other omics. We concluded that, despite of the small sample size available, we had enough power to validate our results and proceed with the analysis.

### Obtaining modules of coordinated metabolites and cytokines

A comparative analysis of several methods including Hierarchical Clustering, K-means and Partitioning Around Medoids (PAM)^30^ was performed and evaluated using a Silhouette analysis^31^. PAM clustering analysis was finally applied to our data since it provided the best Silhouette results. We used the absolute Spearman correlation value (r) between all significant serum features, including metabolites and cytokines. Specifically, distance measure was √2(1–r^2^). Every cluster was a group of features with similar variation patterns across patients and clusters were denoted as “Modules”.

### Integration of multi-omic datasets

We used Partial Least Squares (PLS) regression^32^ to link changes in gene expression with the variation in the serum levels of metabolites and cytokines. PLS integrates two data matrices, X (e.g. transcriptomics) and Y (e.g. metabolomics), maximizing the covariance between them.

For all PLS models, the same set of genes were used as explanatory variables. These genes were selected by having an FDR < 0.05 in the **limma** analysis (i.e. “significant” genes). In this way, we compiled a gene expression matrix of explanatory variables that were relevant to the disease. Then, for each Module-specific PLS model, the serum levels of the metabolites and cytokines included in the model were taken as response Y matrix.

Two parameters were calculated to assess performance of PLS models: R^2^ (multiple correlation coefficient; degree of Y variance explained by X) and Q^2^ (predictive power). As the number of samples *n* is small in this study, leave-one-out cross-validation was used to compute Q^2^. Gene loadings of the resulting PLS models are a measure of the relative importance of the gene variables to the model. Enrichment analysis using **mdgsa** test^33^ were run for the list of genes ranked by the loading values of the first component of the PLS models. The Biological Process branch of the Gene Ontology^34^ was chosen as the annotation database for enrichment analyses.

## Results

### Transcriptomic analysis confirms changes in the immunophenotype in MHE patients and unveils new altered pathways

**Figure 2:**
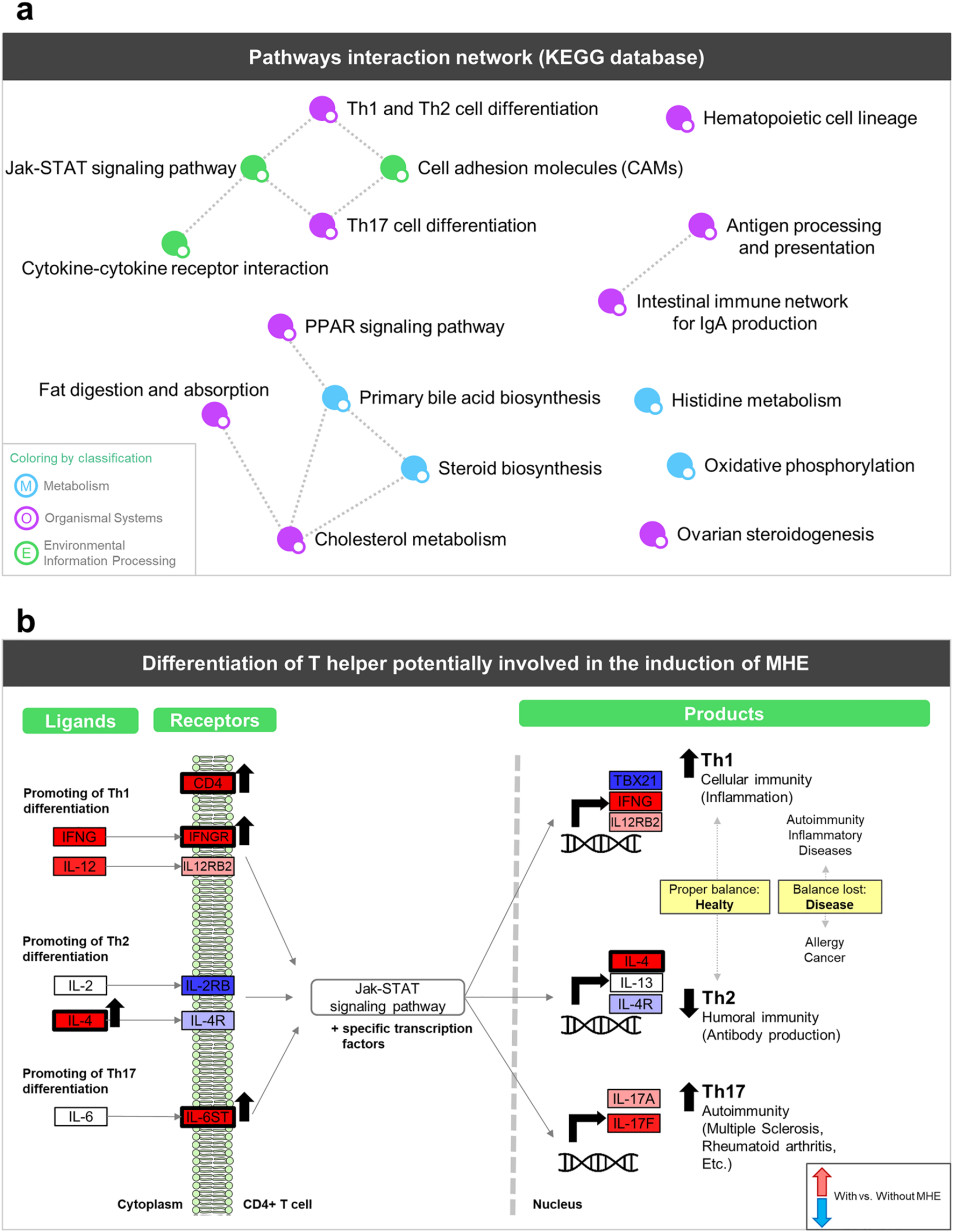
Summary of PaintOmics enrichment analysis. (**a**) Network showing pathway-pathway interactions in PaintOmics 3. Nodes represent pathways with a p-value lower than 0.05 and are colored by KEGG category. Edges between two nodes indicate that both biological processes are closely related. (**b**) Pathway scheme that summarizes Th1, Th2 and Th17 differentiation pathways. Rectangles represent up (red) or down (blue) gene regulation in patients with versus without MHE. Black arrows and thicker black borders mark those genes with statistical significance (FDR < 0.05).

Gene expression analysis identified 847 differentially expressed genes with FDR < 0.05 (Supplementary Table S1) and enrichment analysis showed 11 significant pathways (p-value < 0.05) (Supplementary Table S2) when comparing MHE versus non-MHE patients. Three additional pathways with p-values < 0.1 were included in this selection due to their high relevance to the disease. Pathway enrichment analysis was made with PaintOmics^20^, which creates a network of interconnected significant pathways (Fig. 2A). This analysis revealed a network of immunity related pathways consisting of “Th1 and Th2 cell differentiation” and “Th17 cell differentiation” linked with “Cell adhesion molecules (CAMs)” and “Jak-STAT signaling pathways”, in turn connected with “Cytokine-cytokine receptor interaction”. Jak-STAT pathway plays critical roles in the immune system, especially cytokine receptors and the polarization of T helper cells^35^. In addition, “Antigen processing and presentation” was joined with “Intestinal immune network for IgA production”, while “Hematopoietic cell lineage” remained as a single node. A second group of processes in this network was related to lipid metabolism and included “Fat digestion and absorption”, “Cholesterol metabolism”, “Steroid biosynthesis”, “Primary bile acid biosynthesis” and “PPAR signaling pathway”. This set of functions together with the associated regulated genes depict the most relevant immunological events associated with MHE.

The advantage of using PaintOmics is that this web tool allows the visualization of gene expression levels onto KEGG^21^ pathways. Inspection of the “Hematopoietic cell lineage” pathway revealed significant differences in genes related to B and T cells differentiation (Supplementary Fig. S1). In particular, the mature B-cell marker *MS4A1* (also known as CD20) was up-regulated in MHE patients. This gene is a relevant target for autoimmune disease therapies^36^ and has also been related to T-cell-dependent immune responses^37^. T cells are divided into different subsets according to their markers and functions like CD8+ that are T cytotoxic cells (Tc) and CD4+ are T helper cells (Th). Our results showed a significant up-regulation of *CD4* and significant down-regulation of *CD8A* and *CD8B* in MHE patients. Additionally, the “Antigen processing and presentation” pathway was also generally decreased (Supplementary Fig. S1). Tumor Necrosis Factor (*TNF*) and Proteasome Activator Subunit (*PSME1*), two upstream key genes in Major Histocompatibility Complex (MHC) class I peptides, were significantly down-regulated. *TNF* is able to activate *PSME1* that is implicated in immunoproteasome assembly, required for efficient antigen processing^38^. Moreover, the antigen presentation genes of MHC class II, *HLA-DQA2* and *HLA-DRB5*, were also significantly decreased. Altogether, these results describe an immune response signature in MHE patients where antigen processing and presentation were down-regulated and T cells population showed opposite regulation patterns with Tc down-regulated and Th up-regulated.

More details on changes at T helper lymphocytes were found when analyzing the “Th1 and Th2 cell differentiation” pathway (Fig. 2B). We found significant differences between Th1 and Th2 subtypes in cytokine receptors and products (left and right part of the pathway, respectively). For instance, the Th1-related molecules *IFNGR, IL12R*, *IFNG* and *IL12* were increased while Th2 showed decreased *IL2R* and *IL4R* receptors, significantly increased *IL4* and unaltered *IL13* expression. Th17 lymphocytes are the third Th subset most important for the regulation of immunity^39^ and the “Th17 cell differentiation” pathway showed a significant up-regulation of the *IL6ST* receptor and an increase in *IL17A* and *IL17F* expression that suggested the promotion of Th17 cell type in MHE patients. These new results corroborate previous observations where activity of Th1 and Th17 cells tends to be more elevated in patients with MHE while Th2 cells activity tends to decrease^3^.

We also found significant alterations in the expression of chemokine receptors (*CCR2* and *CXCR3*) and ligands (*CCL5, CXCL5, PF4 (CXCL4), PF4V1 (CXCL4V1)* and *XCL1*) in patients with MHE, which in all cases were down-regulated except for *CCR2*. Chemokine receptor regulation on T cells is a complex mechanism that is dependent on both T cell activation and the differentiation state^40^. For instance, Th1 pro-inflammatory cells preferentially express CCR5, CXCR3 and CXCR6, while the expression of CCR3, CCR4, CCR8 is associated with Th2 anti-inflammatory cells^41–44^. Chemokine receptors such as CCR2 have been proposed to play critical roles in neurological diseases like Alzheimer^45,46^ and Multiple Sclerosis^6^, where it was found to be a critical modulator of the aberrant migration of peripheral T cells towards the site of inflammation. The observed changes in chemokine receptors expression in MHE patients reinforce the hypothesis of a readjustment of the population of immune cell types in these patients.

Beyond immunological processes, also lipid metabolism pathways appeared to be significantly enriched in MHE patients. Differentially expressed genes in this set of pathways were the up-regulated *CD36, ABCA1* (both transporters), *PLPP3, NCEH1* (both enzymes) and the down-regulated apolipoproteins *APOA1* and *APOB*. Indicated differences pointed to an increased fatty acid digestion and absorption in immune cells of MHE patients.

### A coordinated metabolic and cytokines signature is present in MHE patients

We identified 29 metabolites with significant differences between patients with and without MHE, (FDR < 0.05) (see Table 1). Increased metabolites included methionine, 3 sphingomyelins (SMs), 5 phosphatidylcholines (PCs) and octadecenoylcarnitine (C18:2). Metabolites with reduced levels included 17 PCs and 2 lysophosphatidylcholines (lysoPC), spermine, alpha-Aminoadipic (alpha-AAA) acid and valine. The most altered molecules in MHE patients were PCs and lysoPCs, that were mainly down-regulated (negative logFC values). Previously, these phospholipids were found to be decreased in the peripheral blood samples^47^ and postmortem brain samples^48^ of patients with Alzheimer’s disease. As for cytokines, 6 molecules (IL-15, CXCL13, CCL20, CX3CL1, IL-6 and IL-22) were identified as having significant different levels (FDR < 0.05) in patients with or without MHE. All molecules presented increased levels in MHE patients (see Table 2). The majority of cytokines showed the same direction of change at mRNA and protein level in MHE patients (Supplementary Table S3).

**Table 1:** Serum metabolites with significant differences between patients with and without MHE.

ID Biocrates	logFC^a^	FDR^b^	ID Biocrates	logFC^a^	FDR^b^
**PC aa C36:4**	− 0.7436	0.0005	**PC aa C38:5**	− 0.6788	0.0009
**PC aa C38:4**	− 0.7106	0.0006	**PC aa C38:3**	− 0.5105	0.0165
**PC aa C38:5**	− 0.6788	0.0009	**alpha-AAA**	− 0.4547	0.0167
**PC aa C40:5**	− 0.6272	0.0015	**PC aa C40:4**	− 0.4155	0.0228
**PC aa C34:4**	− 0.8185	0.0038	**PC aa C36:6**	− 0.6148	0.0230
**lysoPC a C20:4**	− 0.5669	0.0041	**lysoPC a C20:3**	− 0.6249	0.0245
**PC aa C40:6**	− 0.7801	0.0046	**PC aa C34:3**	− 0.5725	0.0250
**PC aa C38:6**	− 0.8598	0.0051	**Val**	− 0.4825	0.0296
**PC ae C38:0**	− 0.5767	0.0055	**SM C22:3**	1.1125	0.0372
**Met**	0.6004	0.0063	**PC aa C32:3**	− 0.3489	0.0379
**PC aa C36:5**	− 0.8773	0.0067	**PC aa C42:1**	0.3616	0.0386
**SM (OH) C14:1**	0.5611	0.0075	**SM C16:0**	0.3501	0.0399
**PC ae C44:6**	0.6000	0.0137	**PC ae C30:0**	0.4426	0.0409
**Spermine**	− 0.3971	0.0139	**PC ae C42:5**	0.5168	0.0424
**PC aa C36:4**	− 0.7436	0.0005	**PC ae C44:5**	0.5536	0.0473
**PC aa C38:4**	− 0.7106	0.0006	**C18:2**	0.4845	0.0477

Lipids are described with the notation “Cx:y”, where x denotes the number of carbons in the side chain and y denotes the number of double bonds.

*PC* phosphatidylcholine, *lysoPC* lysophosphatidylcholine, *SM* sphingomyelin. alogFC: log2-fold-change; bp-value of t-test calculated and adjusted by FDR with the limma R package.

**Table 2:** Serum cytokines with significant differences between patients with and without MHE.

	IL-6 (pg/ml)	IL-15 (pg/ml)	IL-22 (pg/ml)	CXCL13 (pg/ml)	CCL20 (pg/ml)	CX3CL1 (pg/ml)
**Without MHE**	1.15 (0.13)	4.66 (1.70)	56.04 (5.74)	109.37 (17.82)	58.37 (7.04)	603.38 (79.32)
**With MHE**	3.70 (5.99)	9.71 (2.35)	81.05 (58.09)	194.48 (14.89)	90.20 (10.95)	833.42 (13.75)

Values are median (IQR, Interquartile Range). Differences between patients were tested using non-parametric Wilcoxon test and adjusted by FDR (see “Methods”).

**Figure 3:**
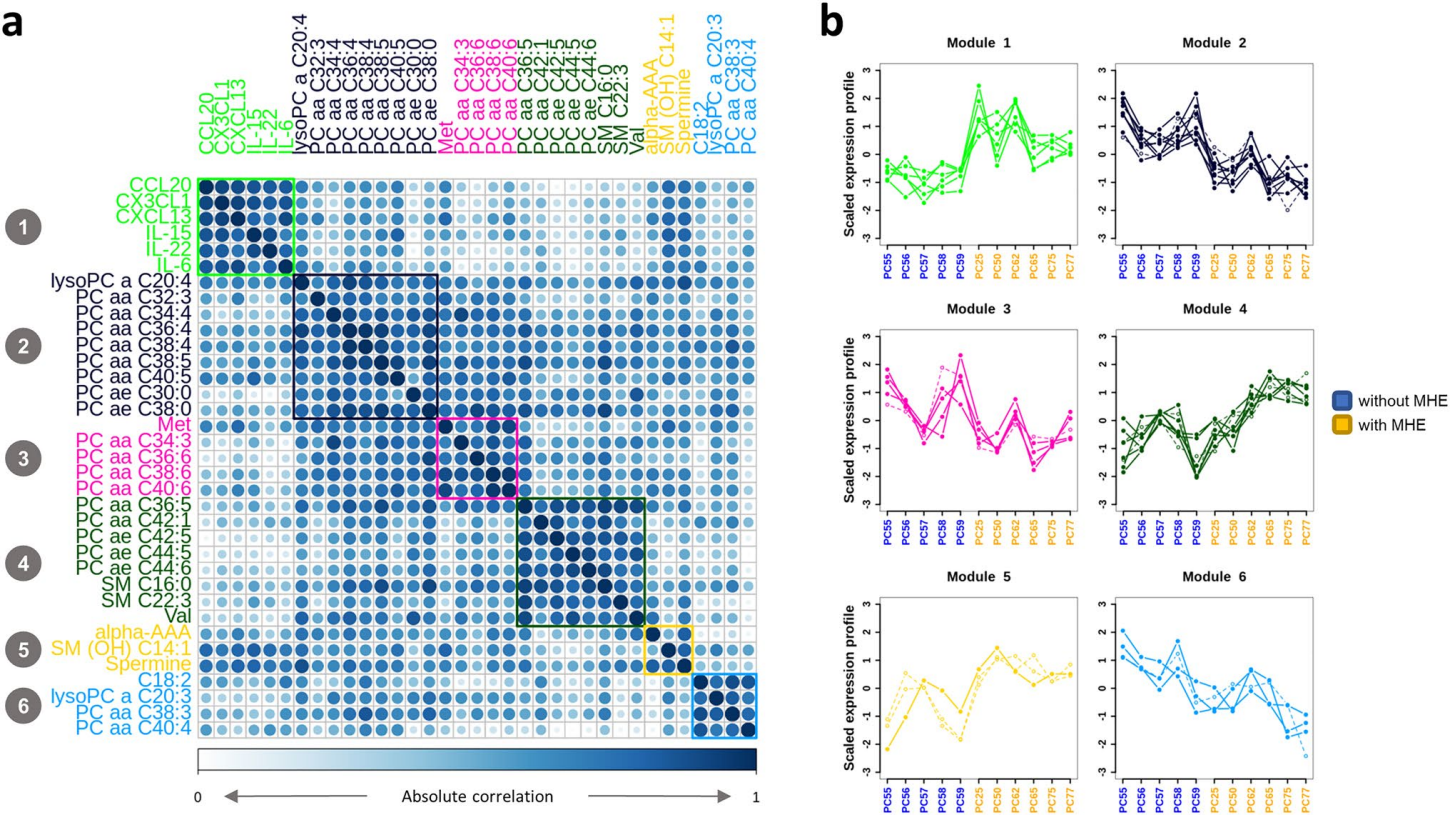
Metabolic and cytokines signature present in MHE patients. (**a**) Absolute correlation plot and clustering analysis of extracellular features. (**b**) Profiles of scaled compound levels across patients indicate the ability to distinguish between the groups of patients with (yellow) and without (blue) MHE. Dashed lines represent compounds with negative correlations with the average block profile, which were set to positive for representation purposes.

We then evaluated the co-abundance patterns of these differential serum compounds across cirrhotic patients. Data clustered into 6 distinct groups where elements were highly correlated (Fig. 3A) and had abundance profiles that distinguished between groups with and without MHE (Fig. 3B). Most correlation groups were composed of chemically similar compounds, suggesting a functional significance for this co-variation pattern. For example, one relevant cluster was composed of cytokines CCL20, CX3CL1, CXCL13, IL-15, IL-22 and IL-6 (Module 1, green in Fig. 3, values shown in Supplementary Table S4), which have been already proposed as candidate modulators of lymphocyte infiltration into the brain and may contribute to cognitive impairment in MHE^3^. In addition, phospholipids almost perfectly clustered by molecular mass (total number of carbon atoms) and saturation level (total number of double bonds) in 3 main functional modules: (a) short and softly unsaturated lipids with ≤ 40C and ≤ 5 double bonds (purple Module); (b) short and highly unsaturated lipids with ≤ 40C and > 5 double bonds (pink module); (c) long and softly unsaturated lipids with > 40C and ≥ 5 double bonds (dark green Module).

### Multi-omic integration analysis highlights gene pathways related with metabolites and cytokines altered in MHE patients

**Figure 4:**
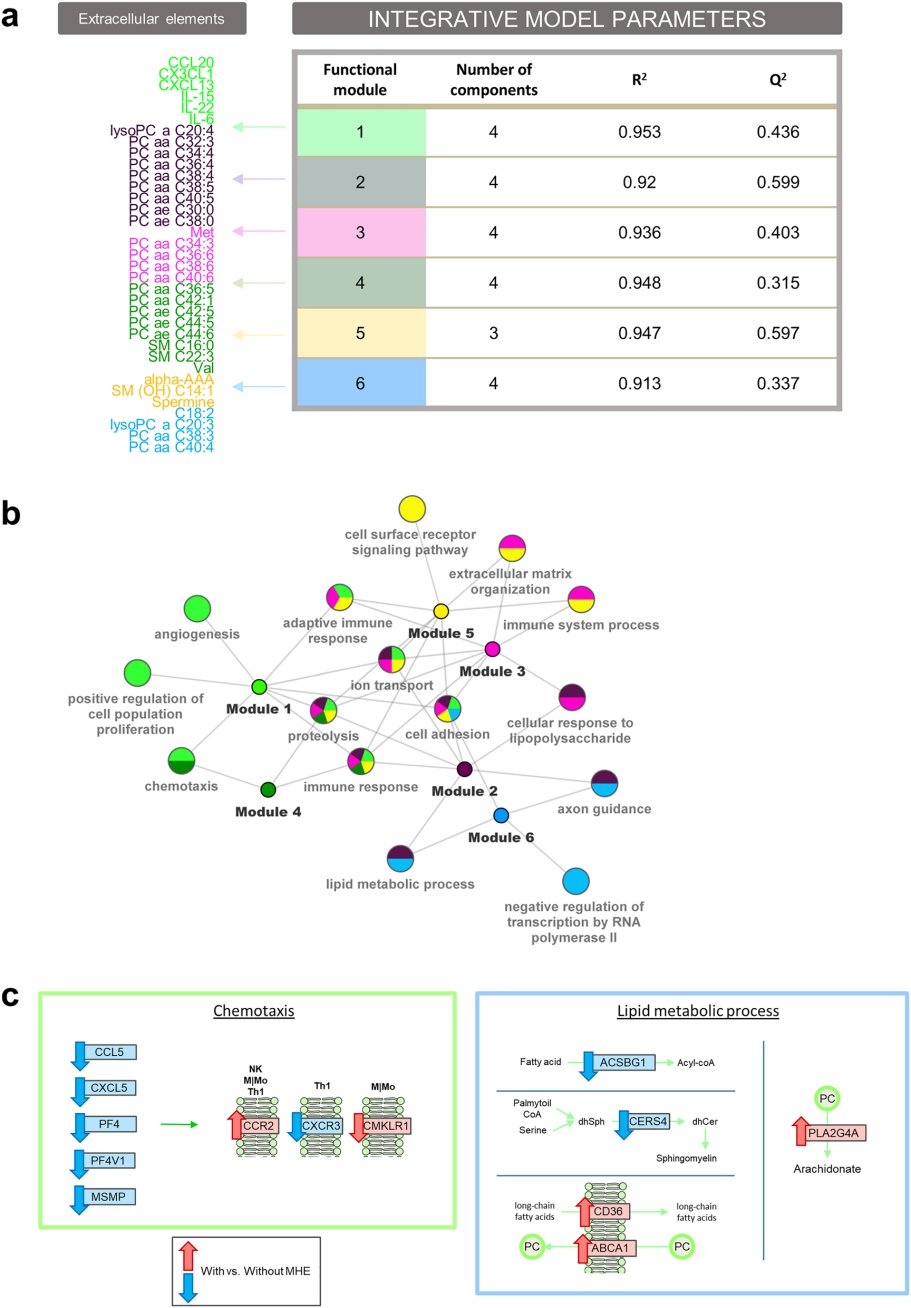
Multi-omic integration analysis and biological interpretation. (**a**) Results of PLS integrative models. R^2^: goodness of fit. Q^2^: goodness of prediction. (**b**) Multi-enrichment network. Every node is a selected significant GO biological process from the enrichment analysis in the PLS models. Edges denote links to the metabolites/cytokines modules where these pathways were found to be significant. (**c**) Some MHE altered GO terms at gene (rectangles) and metabolite (circles) level. Arrows indicate significant up (red) or down (blue) regulation in patients with versus without MHE.

The previous analyses identified transcriptional signature in whole blood cells and a metabolic signature in serum that is associated with MHE. We then asked whether both signatures could be related by identifying gene expression signals that correlated with the extracellular compound changes linked to cognitive impairment. To answer this question, we established Partial Least Square (PLS) multivariable regression models where differentially expressed genes were taken as explanatory variables, and each of the 6 metabolic modules were used as joint response variables to obtain a model per module. The accuracy and predictive ability of these models were determined by the goodness of fit (R^2^) and goodness of prediction (Q^2^), respectively. All 6 PLS models had high (> 0.9) R^2^, indicating a good explanatory value, while most models had good (> 0.5) Q^2^ predictive values (Fig. 4A).

One interesting property of the PLS method is the direct interpretability of the loading values as a measure of the relative importance of the variables in the model. To further understand the biological significance of the models, gene set enrichment tests were run for genes ranked by their loading values at the first component, which provided the best separation between patients with and without MHE in each of the six models (Supplementary Fig. S2). Results were summarized in a multi-enrichment graph (Fig. 4B), where every node is a selected GO term from the enrichment analysis of PLS loadings and the edges link them to the compound modules in which they were found to be significant. This representation locates module-specific pathways at the periphery of the network, while shared pathways are grouped towards the center. Overall, frequently shared pathways included “cell adhesion”, “immune response”, “proteolysis”, “ion transport” and “adaptive immune response”, while module-specific pathways involved distinct signaling and metabolic pathways. Details of the significant genes within each enriched pathway are given in Supplementary Table S5.

Interestingly, the model for Module 1, that consisted of the previously highlighted cytokines CCL20, CX3CL1, CXCL13, IL-15, IL-22 and IL-6, showed enrichment for 3 immune related pathways including “chemotaxis”, “adaptive immune response” and “immune response” (Fig. 4B). At the “chemotaxis” pathway, significant alterations in the expression of chemokine receptors (CCR2, CXCR3 CMKLR1) and ligands (CCL5, CXCL5, PF4 (CXCL4), PF4V1 (CXCL4V1) and MSMP) were found in patients with MHE (Fig. 4C), which in all cases were down-regulated except for CCR2 and CMKLR1 (values shown in Supplementary Table S4). These results indicate the connection between the cellular chemotactic response discussed above and the serum accumulation of inflammatory markers.

Another interesting model was obtained for the Module 2, composed of phosphatidylcholines with ≤ 40 carbons and between 0 and 5 double bond in the fatty acid chain, that appeared correlated to genes of the “lipid metabolic process”, “cellular response to lipopolysaccharide” and “immune response”, among other pathways (Fig. 4B). The lipid metabolic process pathway was shared also by Module 6 (composed of similar phospholipid in length and saturation level) and included annotated genes from different pathways. In particular, we identified the down-regulation of *ACSBG1* and *CERS4* (Fig. 4C). *ACSBG1* catalyzes the conversion of very long-chain fatty acids into their active form (acyl-CoA) for both biosynthesis and degradation of cellular lipids^49^, while *CERS4* catalyzes formation of SMs with high selectivity toward long and very-long chains^50^. This result suggests that impairment of long-chain fatty acids activation and SM production may be on-going in MHE patients. Additionally, we found two significantly up-regulated lipid transporters within this pathway: *ABCA1* (transport phosphatidylcholine from cell to environment^51^) and *CD36* (internalization of long-chain fatty acids into cells^52^), pointing to an increased fatty acid transport in MHE. CD36 is also a scavenger receptor that cooperates with Toll-Like receptors in the internalization of oxidized phospholipid activating the inflammasome in macrophages^53^. Moreover, the gene *PLA2G4A* was up-regulated in MHE patients its gene product selectively hydrolyzes phosphatidylcholines in arachidonic acid that finally derives in prostaglandins, and together with its enzymatic activity, it is also implicated in the initiation of the inflammatory response^54^.

## Discussion

This work identified immune system pathways potentially involved in the induction of MHE by integrating multiple molecular assays from human blood samples. Transcriptomic analysis suggested new altered immune subtypes in MHE patients like up-regulated B-cells and down-regulated cytotoxic CD8 T (Tc) cells. It has been proposed that the appearance of MHE is associated to a shift in peripheral inflammation to an autoimmune-like profile^3^. The up-regulation of B cells may contribute to potentiate this shift. It has been reported that B cells can contribute to autoimmune diseases through different functions such as secretion of autoantibodies, presentation of autoantigen, secretion of inflammatory cytokines, modulation of antigen processing and presentation or generation of ectopic germinal centers^55^. The up-regulation of B cells in MHE may induce some of these functions and contribute to triggering the appearance of MHE.

CD8+ T cell deficiency and an increased CD4/CD8 ratio are features of many human chronic autoimmune diseases^56^. It is also an early and persistent feature of patients with multiple sclerosis. Deficiency of CD8+ T cells is present at the onset of multiple sclerosis and persists throughout the clinical course^57^. The reduced total CD8+ T cells in the blood of these patients has been attributed to sequestration of CD8+ T cells in the brain^58^. It is therefore likely that an early down-regulation of CD8+ T cells could also contribute to trigger the autoimmune shift in peripheral inflammation of MHE patients and to promote infiltration of T cells into the brain.

The results reported here also confirmed the inflammatory signature previously detected in MHE individuals and suggested cytokine signaling pathways as putative mechanisms for the decrease of Th2 and the increase of Th1 and Th17 cells in MHE patients. In a previous study, using isolated CD4+ lymphocytes in culture, we identified in the medium higher levels of IL-17, IL-21, IL-22 and TNFα(3), which are a signature of factors released by for Th17 and Th22 cells. This supports increased activation of these Th-cell subtypes and further validates the results of our analysis approach. Th17 cells are one of the major pathogenic Th cell populations underlying the development of many autoimmune diseases, including multiple sclerosis, rheumatoid arthritis or psoriasis^59^. The increase in Th17 would also contribute to promote an autoimmune profile in patients with MHE. This shift to autoimmune-like inflammation in turn would trigger mild cognitive impairment.

We have also identified other yet unexplored biological processes such as lipid metabolism to play important roles in the pathogenesis of MHE. The PLS analysis also revealed that long-chain unsaturated phosphatidylcholines, increased fatty acid transport and prostaglandin production are strongly linked in MHE patients, suggesting a possible pathway for the dysregulation of structural phospholipids during this mild cognitive decline. Altered lipid metabolism in immune system cells has also been found in other diseases associated to cognitive impairment such as Alzheimer’s disease^60^ and Mild Cognitive Impairment^61^. Notably, the relationship between phospholipids and peripheral inflammation has been extensively reported in the context of autoimmune diseases^62,63^. For instance, metabolomic measures using the same technological platform used here, identified a panel of lipids that predict mild cognitive impairment or Alzheimer’s disease^64^ and three of them, PC aa C36:6, PC aa C38:6, PC aa C40:6, were also significantly down-regulated in MHE patients. Another lipidomic screen in Alzheimer’s disease^47^ identified 3 phosphatidylcholine molecules that were significantly diminished in cases. The authors hypothesized that altered phospholipase (PLA2) activity could lead to an increased metabolism of the PCs, and thereby a subsequent decrease in plasma levels. This is part of the Lands cycle for synthesis and degradation of PCs, where plasma PCs are synthesized in the liver and secreted as components of lipoprotein particles. PCs are hydrolyzed by PLA2 producing lysoPC, which is rapidly cleared from circulation by transporters to the liver for the synthesis of PC, closing the cycle^65^. In agreement, our study shows an increase of PLA2G4A in the immune system cells of MHE patients which may contribute to the decrease of PCs. It is relevant to note that a differential phospholipid pattern is detected in peripheral blood of patients having or not cognitive decline in MHE as well as in Alzheimer’s disease^47^ and Parkinson^66^.

During inflammation or under conditions of oxidative stress, the polyunsaturated fatty acid side chains of phospholipids in lipoproteins or cellular membranes can be oxidatively modified, generating structurally diverse oxidized phospholipid (OxPL) species and reactive aldehydes [e.g. malondialdehyde (MDA), hydroxynonenal (HNE)]^67^, each of which may exert both pro-inflammatory and anti-inflammatory effects. We showed in previous studies that patients with MHE had increased oxidative stress in blood compared with cirrhotic patients without MHE. Furthermore, MDA, an indicator of oxidative damage to lipids, was also increased in patients with MHE and correlated with psychometric tests^68^. Altered lipid metabolism is here described for the first time in the context of mild cognitive impairment in MHE. The mechanisms by which these changes in lipids contribute to MHE could be similar to those involved in mild cognitive impairment^64^ and Alzheimer's disease^69^.

Another relevant contribution of this study is the identification of specific sets of serum metabolites and cytokines. The majority of cytokines showed the same direction of change at mRNA and protein level in MHE patients (Supplementary Table S3). Since cytokine proteins were measured in plasma while mRNA was obtained from blood cells, a perfect correlation between both levels was not expected. Firstly, mRNA for cytokines are subjected to post-transcriptional regulation by RNA binding proteins^70,71^ or microRNAs^72^. In addition, plasma cytokines can derive from sources other than blood cells. For instance, IL-15, CX3CL1, IL-17 and TNFα can come from the liver^73–75^. Peripheral lymphocyte subtypes Th17 and Th22 may infiltrate into the liver with subsequent IL-17 and TNFα production, which may contribute to the up-regulation at plasma level of these cytokines in MHE patients^75^.

Our findings that MHE-regulated immune response processes relate to changes in specific serum molecules point to a chemotactic function or structural patterns of lipids as mediators of MHE induction. Integrative modelling suggests a link between certain cytokines and the alteration of chemotactic receptors and ligands in MHE. This reveals a link between the production and reception of inflammatory signals operating in these patients which may contribute to infiltration of peripheral blood cells into the brain and triggering of MHE. The model for Module 1, consisting of the cytokines CCL20, CX3CL1, CXCL13, IL-15, IL-22 and IL-6, showed enrichment for 3 immune related pathways including “chemotaxis”, “adaptive immune response” and “immune response”, indicating the connection between the cellular immune response and the serum accumulation of inflammatory markers. Further inspection of the “chemotaxis” GO term revealed that this extracellular module of cytokines was related with the chemokine receptors and ligands (CCR2, CXCR3, CCL5, CXCL5, PF4 and PF4V1). Many of these chemokine receptors are altered in multiple sclerosis patients, who show high levels of CCR2, CCR5, CXCR3^76^. Similarly, mouse models of inflammatory liver injury showed increased numbers of circulating CCR2-expressing monocytes that are attracted to the brain by activated microglia^77^, while in vitro models of transmigration revealed that T cells from multiple sclerosis patients exhibit an increased attraction to CCL3 and CCL5^78^. Moreover, the ligand MSMP acts as ligand for CCR2 and exhibits a chemotactic activity for monocytes and lymphocytes^79^ while the receptor CMKLR1 plays a key role in directing plasmacytoid dendritic cell trafficking^80^. In a similar way, these results suggest an important role of chemokines in MHE patients related to trafficking of peripheral blood cells into the brain already shown in MHE^4^.

In summary, this work identified immune system pathways potentially involved in the induction of MHE by integrative multi-omic analysis of multiple molecular parameters from human blood samples. We detected biological pathways that corroborate the observed decrease of Th2 and the increase of Th1 and Th17 in MHE patients, while other pathways suggested B-cell up-regulation and CD8 T cells down-regulation. Lipid metabolism is identified for the first time as related with mild cognitive impairment in MHE. Secondly, specific sets of serum metabolites and cytokines were identified that point to chemotactic function or structural patterns of lipids as mediators of MHE induction. Finally, integrative modelling suggests a link between cytokines CCL20, CX3CL1, CXCL13, IL-15, IL-22 and IL-6 and the alteration of chemotactic receptors (CCR2, CXCR3, CMKLR1) and ligands (CCL5, CXCL5, PF4, PF4V1 and MSMP) in MHE. Our integrated model is able to link the extracellular information (metabolites/cytokines) with the gene expression in blood human samples of MHE patients. These results provide the basis for further studies on the mechanisms by which changes in peripheral inflammation trigger mild cognitive impairment in patients with liver cirrhosis and MHE. Our results also illustrate the power of the integrative statistical analysis of multi-omics in modelling disease processes and connecting phenotypic changes across molecular layers. For diseases where multiple underlying mechanisms are involved in pathogenesis like MHE^81^, multi-component analysis increases the chances of detecting relevant molecular signals that can be combined for diagnosis. Our pilot study (n = 11) suggests that indeed alterations in MHE patients occur in different types of biomolecules, which are functionally connected and have the potential to serve as biomarkers for the disease. However, future work is required to validate these results with a larger cohort and establish the utility of these molecular mechanisms as biomarkers of MHE.

## Supplementary Information

Supplementary Information 1.

Supplementary Information 2.

## Data Availability

The transcriptomic dataset supporting the conclusions of this article is available in the GEO database repository, GSE149741, https://www.ncbi.nlm.nih.gov/geo/query/acc.cgi?acc=GSE149741. The rest of datasets supporting the conclusions of this article are included within the article (Supplementary Tables S6 and S7).
