# Supplementary material for: Multi-omic analysis unveils biological pathways in peripheral immune system associated to minimal hepatic encephalopathy appearance in cirrhotic patients
Source: Sci Rep. 2021 Jan 21;11:1907. doi: 10.1038/s41598-020-80941-7 (PMC7820002; doi:10.1038/s41598-020-80941-7)
Supplement: Supplementary file 1 — Supplementary Information 1. [file 41598_2020_80941_MOESM1_ESM.pdf]

Supplementary information

Multi-omic analysis unveils biological pathways in peripheral immune system associated to minimal hepatic encephalopathy appearance in cirrhotic patients

Teresa Rubio[1,9], Vicente Felipo[1,9], Sonia Tarazona[2,9], Roberta Pastorelli[3], Desamparados Escudero-García[4], Joan Tosca[5], Amparo Urios[1,6], Ana Conesa[7, *], Carmina Montoliu[6,8]

1 Laboratory of Neurobiology, Centro Investigación Príncipe Felipe, Valencia, Spain.

2 Departamento de Estadística e Investigación Operativa Aplicadas y Calidad, Universitat Politècnica de València, Valencia, Spain.

3 Protein and Metabolite Biomarkers Unit, Laboratory of Mass Spectrometry, Istituto di Ricerche Farmacologiche Mario Negri IRCCS, Milano, Italy.

4 Unidad de Digestivo, Hospital Clínico de Valencia. Departamento Medicina, Universidad de Valencia, Valencia, Spain.

5 Unidad de Digestivo, Hospital Clínico de Valencia, Valencia, Spain.

6 Neurological Impairment Laboratory. Fundación Investigación Hospital Clínico Universitario de Valencia. Instituto de Investigación Sanitaria-INCLIVA, Valencia, Spain.

7 Microbiology and Cell Science Dep, Institute for Food and Agricultural Sciences, University of Florida, Gainesville, USA.

8 Departamento de Patología, Facultad de Medicina, Universidad de Valencia, Spain.

9 These authors contributed equally: Teresa Rubio, Vicente Felipo and Sonia Tarazona.

* email: aconesa@ufl.edu

Supplementary Figure S1. Heatmap of relevant genes involved on two significant pathways of MHE.

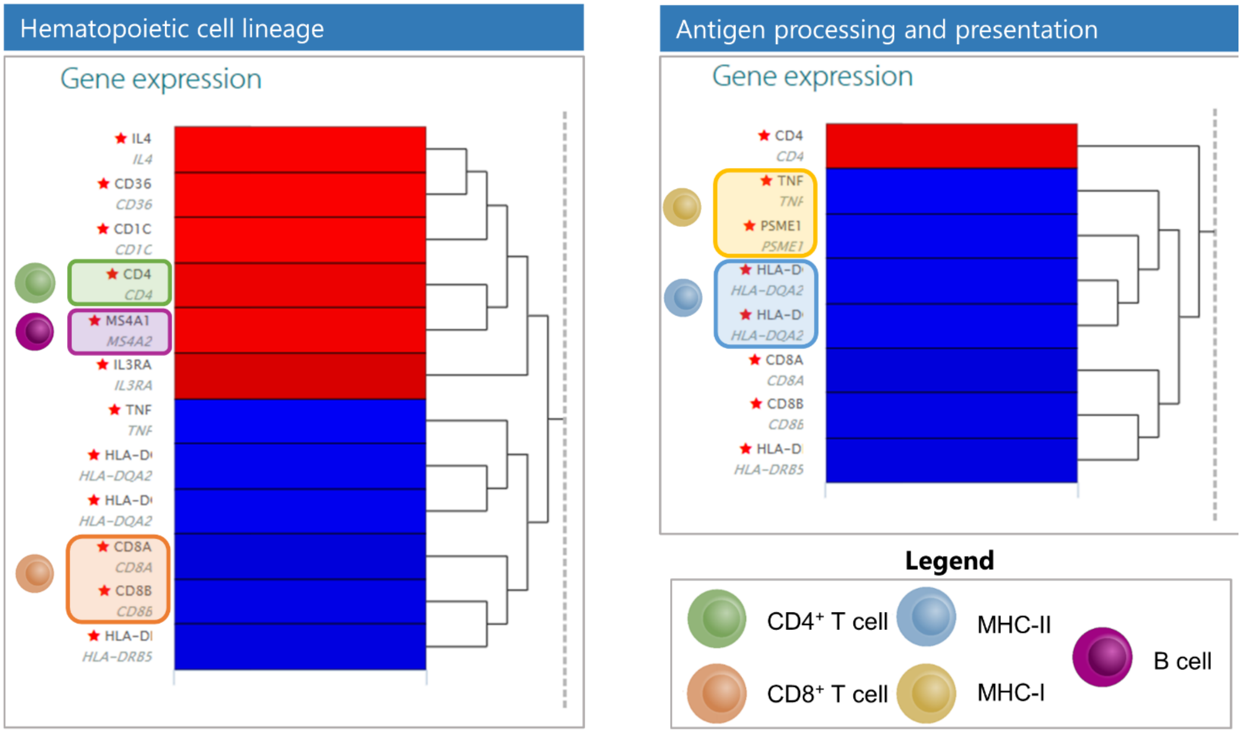

Figure S1: Red and blue boxes represent respectively significant up- and down-regulation at gene expression level in patients with MHE versus without MHE. Gene markers of specific immune cell subtypes are highlighted in different colors according to legend pattern.

Supplementary Table S3. Cytokine direction of change in MHE at mRNA and protein level.

	CYTOKINES DATASET			Microarray dataset			Coincidence
	**FDR**	**logFC**	**Regulation**	**FDR**	**logFC**	**Regulation**	
**CCL20**	0.02	0.63	UP	NA	NA	NA	?
**CX3CL1**	0.02	0.47	UP	0.23	-0.35	DOWN	NO
**CXCL13**	0.02	0.83	UP	NA	NA	NA	?
**IL-10**	0.13	0.35	UP	0.13	0.43	UP	YES
**IL-12**	0.4	0.18	UP	0.12	0.3	UP	YES
**IL-13**	0.77	-0.16	DOWN	NA	NA	NA	?
**IL-15**	0.02	1.06	UP	0.36	-0.2	DOWN	NO
**IL-17**	0.35	0.43	UP	0.93	-0.02	DOWN	NO
**IL-18**	0.28	0.31	UP	0.37	0.21	UP	YES
**IL-22**	0.02	0.53	UP	NA	NA	NA	?
**IL-4**	0.92	0	UP	0.01	0.66	UP	YES
**IL-6**	0.02	1.68	UP	0.89	0.04	UP	YES
**TGF-B**	0.09	-0.68	DOWN	0.27	-0.25	DOWN	YES
**TNF-a**	0.12	0.75	UP	0.02	-0.45	DOWN	NO

Table S3: The table shows significance (FDR) and log fold-change (logFC) between patients with MHE and without MHE in both cytokine and transcriptomics datasets extracted from their respective statistical tests. Some cytokines were not represented in the Agilent microarray (represented by NA).

Supplementary Table S4. Changes of some genes and cytokines between patients with and without MHE.

MODULE1 (cytokines)	withoutMHE	withMHE	logFC
CCL20	58.37 (7.04)	90.2 (10.95)	0.63
CX3CL1	0.6 (0.08)	0.83 (0.14)	0.47
CXCL13	109.37 (17.83)	194.48 (14.89)	0.83
IL-15	4.66 (1.7)	9.72 (2.36)	1.06
IL-22	56.04 (5.74)	81.05 (58.09)	0.53
IL-6	1.15 (0.13)	3.7 (5.99)	1.68
**Chemotaxis pathway (genes)**	**withoutMHE**	**withMHE**	**logFC**
CCL5	13.89 (0.25)	13.34 (0.8)	-0.55
CXCL5	8.07 (0.96)	7.34 (0.59)	-0.73
PF4	14.01 (0.38)	13.2 (1.27)	-0.81
PF4V1	8.28 (0.52)	6.41 (1.37)	-1.87
MSMP	4.51 (0.43)	4.16 (0.5)	-0.35
CCR2	10.51 (0.33)	11.19 (0.84)	0.68
CXCR3	11.05 (0.38)	10.45 (0.48)	-0.6
CMKLR1	6.05 (0.86)	6.96 (1.02)	0.91

Table S4: Values for each group (withoutMHE and withMHE) are the median and interquartile range between parenthesis. Note that gene expression is in log2 scale after vsn transformation while cytokines are raw concentration data. Log Fold Change (logFC) is calculated as log2(withMHE / withoutMHE).

Supplementary Figure S2. Score plots of the six PLS models.

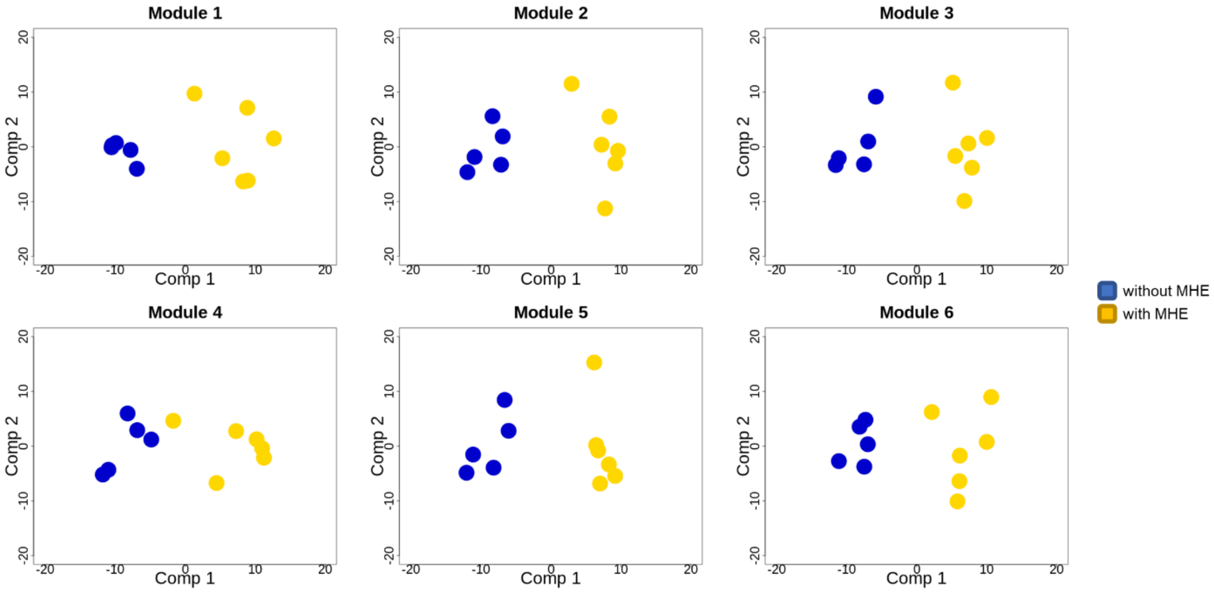

Figure S2: Component 1 achieves the best separation between patients with and without MHE in each of the six models. Blue represents patients without MHE and yellow patients with MHE.
